# Influence of genetic substructuring of statistical forensic parameters on genetic short tandem repeat markers in the populations of Southeastern Europe

**DOI:** 10.3325/cmj.2022.63.244

**Published:** 2022-06

**Authors:** Natalija Novokmet, Marijana Peričić Salihović, Vedrana Škaro, Petar Projić, Jelena Šarac, Dubravka Havaš Auguštin, Pavao Rudan, Dragan Primorac, Damir Marjanović

**Affiliations:** 1 Institute for Anthropological Research, Zagreb, Croatia; 2 DNA Laboratory, Genos Ltd., Zagreb, Croatia; 3 Laboratory for Molecular Anthropology, Center for Applied Bioanthropology, Institute for Anthropological Research, Zagreb, Croatia; 4 Scientific Council for Anthropological Research, Croatian Academy of Sciences and Arts, Zagreb, Croatia; 5 St. Catherine Hospital, Zagreb, Croatia; 6 School of Medicine, University of Split, Split, Croatia; 7 University Department of Forensic Sciences, University of Split, Split, Croatia; 8 School of Medicine Rijeka, University of Rijeka, Rijeka, Croatia; 9 Faculty of Medicine, University of Osijek, Osijek, Croatia; 10 Faculty of Dental Medicine and Health, University of Osijek, Osijek, Croatia; 11 Eberly College of Science, Penn State University, University Park, PA, USA; 12 Henry C. Lee College of Criminal Justice and Forensic Sciences, University of New Haven, West Haven, CT, USA; 13 Medical School REGIOMED, Coburg, Germany; 14 The National Forensic Sciences University, Gandhinagar, Gujarat, India; 15 Department of Genetics and Bioengineering, International Burch University, Sarajevo, Bosnia and Herzegovina

## Abstract

**Aim:**

To investigate the influence of specific intrapopulation genetic structures on interpopulation relationships. Special focus was the influence of island population isolation on the substructuring of the Croatian population, and the influence of regional population groups on the substructuring of Southeast European populations.

**Methods:**

Autosomal short tandem repeat (STR) loci were analyzed by using four forensic parameters: matching probability (PM), power of discrimination (PD), power of exclusion (PE), and polymorphic information content (PIC) on a sample of 2877 unrelated participants of both sexes. A sample set comprising 590 participants was analyzed for the first time, and 2287 participants were included from previous studies. The analysis was performed with PowerStats v. 1.2.

**Results:**

The analysis of forensic parameters for all nine loci in the Croatian subpopulations showed the largest deviations in the populations of the islands of Korčula and Hvar. The smallest deviations were found in the mainland population. As for Southeast European populations, the largest deviations were found in the population of North Macedonia, followed by Romania, Albanians from Kosovo, and Montenegro, while the smallest deviations were found in the population of Hungary.

**Conclusion:**

The comparison of forensic parameters between different subpopulations of Croatia and Southeast Europe indicates that the isolation of individual Croatian subpopulations and rare alleles in their gene pool affect the values of forensic parameters. Specific features of (sub)populations should be taken into account for appropriate sampling of the total population when creating a DNA database of STR markers.

Microsatellite markers are used in forensic research due to their high power of discrimination (PD) (usually >0.9, with observed heterozygosity >70%), position at separate chromosome locations (to avoid closely related loci), consistency and reproducibility of results after multiplication with other markers, and rare occurrences of stutter products (1). The use of short tandem repeat (STR) markers in paternity testing and forensic genetic analysis increases the PE from an initial 30% to 40% for blood typing, 80% for tissue typing, HLA analysis, 90% for combinations of HLA analysis with serological tests, and finally 99.99% for RFLP analysis to a minimum of 99.999% (2-4). In forensic research, the advantage of microsatellite markers is the possibility of simultaneous analysis of multiple loci in multiplex STR systems, which allows a high degree of individualization in identifying traces. In forensic applications where DNA is usually degraded, microsatellite markers of 100 to 400 bp are better markers than minisatellites (VNTRs) of 400 to 1000 bp (4).

Microsatellite DNA differs for each person, but two people may have the same allelic variants at one or more STR loci. However, the probability of finding allelic variants in two individuals at, for example, 15 STR loci, analyzed using the PowerPlex 16 kit for the white population is 1.83 × 10^17^ (5). The average mutation rate for loci is variable, but its values are below 0.1%. Based on previous forensic research, the loci with the lowest mutation rate are CSF1PO, TH01, TPOX, D5S818, and D8S1179, while D21S11, FGA, D7S820, D16S539, and D18S51 have a significantly higher mutation rate (2,6-8).

Specific population DNA databases serve to determine the genetic diversity of populations and to facilitate statistical calculations in forensic genetics. They are not based on individual DNA profiles, but the profiles represented in a particular population are used to determine allelic frequencies for further statistical calculations, ie, analysis of forensic identification parameters based on the assessment of PM, PD, PE, degree of loci heterozygosity (H), and likelihood ratio (LR). In order to form the most representative database of a certain population, it is important to investigate the genetic diversity of the population, its features, size, isolation, and the degree of genetic differentiation in relation to neighboring and distant populations (4).

Therefore, the aim of this study was to investigate the existence of substructured subpopulations in Croatia, and the impact of their specific intrapopulation genetic structure on interpopulation relationships, and how these relationships affect the basic forensic statistical parameters of genetic STR markers.

## Methods

### Sample

A total of 2877 unrelated participants of both sexes from the area of Southeastern Europe were analyzed. A sample set comprising 590 participants was analyzed for the first time in this study and includes the following populations: Baranja (n = 397), island of Ugljan (n = 58), island of Pašman (n = 10), island of Dugi Otok (n = 14), and Slovenia (n = 111). The islands of Ugljan, Pašman, and Dugi otok were analyzed as one population (north Dalmatian islands, NDI) due to the small number of samples and relatively small geographical distance and good connections between them. Blood samples of all participants were collected as part of field research, after participants signed the informed consent. DNA isolation was performed in the Laboratory for Molecular Anthropology of the Institute for Anthropological Research, Zagreb, and the genetic analyses of STR markers were performed in the DNA Laboratory, Genos Ltd, Zagreb.

The second set (data for 952 participants) of samples were samples from the biobank of the Institute for Anthropological Research, collected during field research. The set consisted of the following (sub)populations: continental Croatia (Zagreb, Pazin, Delnice, Zabok, and Donji Miholjac, n = 100) (9), island of Cres (n = 122) (10,11), island of Krk (n = 137) (12), island of Brač (n = 96) (13), island of Hvar (n = 103) (14), island of Korčula (n = 95) (15), island of Vis (n = 98) (9), Montenegro (n = 101) (16), and North Macedonia (n = 100) (17).

The third set (data for 1335 participants) was from the available European database with available row data, and consisted of the following populations: Serbia (n = 356) (18), Hungary (n = 223) (19), Bosnia and Herzegovina (n = 100) (20), Romania (n = 222) (21), Kosovo (Albanians) (n = 137) (22), and Greece (n = 297) (23).

To enable a hierarchically structured analysis, the entire sample was divided into two hierarchical groups. The first group consisted of all Croatian subpopulations: mainland (Baranja and continental Croatia) and islands: Krk, Cres, NDI, Brač, Hvar, Korčula, and Vis. The second group consisted of Southeast European populations (Montenegro, Serbia, Croatia, North Macedonia, Hungary, Bosnia and Herzegovina, Romania, Albanians from Kosovo, Greece, and Slovenia).

In order to determine the relationship between the forensic parameters of the Croatian subpopulations, a “potential” reference base of the Croatian population was simulated. Out of 1230 respondents, a sample including 45% of the participants was formed by random selection. The “potential” reference base of the population of Southeast Europe was simulated using the same principle. Out of 1805 participants, a sample for the “potential” reference base was formed including 30% of participants. The sample of Croatia, which included 1230 respondents in the hierarchical level analysis, was reduced to 158 participants to mirror their actual share among Southeast European populations for which microsatellite databases were used in the study (the ratio of continental and island populations in Croatia is 97.2% to 2.8%). Specifically, the Croatian sample included the continental part (n = 100) (Zagreb, Pazin, Delnice, Zabok, and Donji Miholjac) (9), a randomly selected sample from Baranja (N = 44), and 5 samples from Baranja with DNA profiles containing alleles characteristic only for the Croatian population. The island part contained 9 samples selected randomly or based on their unique presence in the Croatian population. In this way, the ratio of continental to island populations was 94.3% to 5.7%, presenting a slightly higher percentage in favor of the islands because of the inclusion of their specific DNA profiles without which the Croatian population base would not be representative.

### STR marker analysis

DNA was isolated from whole-blood samples by using the salting out method (24), followed by polymerase chain reaction (PCR) and capillary electrophoresis. AmpFLSTR Identifiler PCR Amplification Kit (Applied Biosystems, Foster City, CA, USA) was used for STR genotyping. Fragment analysis was performed on 3130 genetic analyzer (Applied Biosystems), ABI Data Collection Software, and GeneMapper^TM^ 3.2 Software (Applied Biosystems).

### Bio-statistical analyses

Since complete data for 2877 samples were not available for all loci included in the AmpFLSTR Identifiler PCR Amplification Kit, in order to make the data comparable, bio-statistical analyses were performed for nine autosomal STR loci (D3S1358, vWA, FGA, TH01, TPOX, CSF1PO, D5S818, D13S317, D7S82).

### Forensic parameters

The following forensic parameters were used in the study: i) matching probability or probability of match (pM), which shows how many people need to be searched to find the same DNA profile in a randomly selected individual; ii) discrimination power (Pd), which is calculated by subtracting pM (1-pM) from the number 1, iii) exclusion power (PE), which is defined as the proportion of individuals whose DNA profile differs from a randomly selected individual in a typical paternity case, and iv) the degree of heterozygosity (H), or a measure of standard genetic diversity. The informativeness of genetic markers increases with increasing degree of heterozygosity. However, when the sample was collected from a genetically isolated population, a lower total number of alleles and a smaller number of alleles per locus can be expected to occur than when collected from a genetically heterogeneous population. Although the degree of heterozygosity increases with increasing number of alleles, it depends on its frequency. Regardless of the number of alleles at a locus, the degree of heterozygosity is maximal when the frequencies of the alleles are equal. Another forensic parameter used in the analysis is the degree of polymorphism (PIC), another measure of the informativeness of genetic markers. The analysis of all the forensic parameters was performed by using the statistical package PowerStats v1.2 (25).

### Data standardization (Z-values)

In order to compare the forensic parameters of the subpopulations of Croatia and the population of Southeast Europe through the differences of the mean values for real and “simulated” samples, the original values were transformed into standardized or Z-values. The standardized value was calculated by determining the deviation of an entity from the arithmetic mean. Thus, the standardized value is a relative measure of deviation of each entity from the arithmetic mean expressed in parts of the standard deviation (26).

## Results

### Forensic parameters in Croatian subpopulations

**Figure 1:**
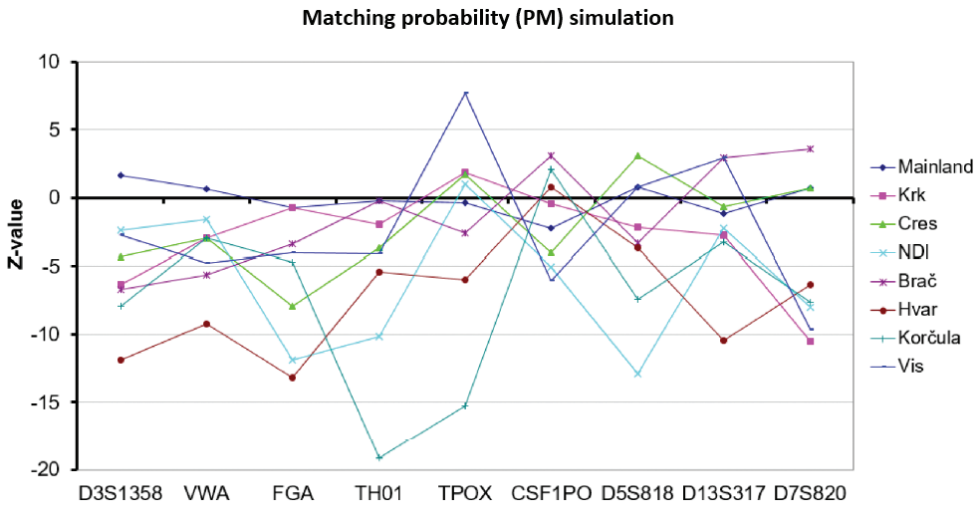
Matching probability (PM) Z-values in the subpopulations of Croatia in relation to the simulated population. NDI – North Dalmatian ilands.

**Figure 2:**
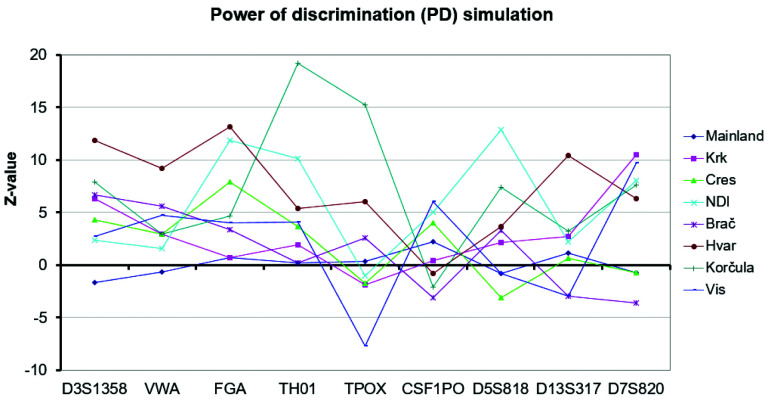
Power of discrimination (PD) Z-values in the subpopulations of Croatia in relation to the simulated population. NDI – North Dalmatian islands.

**Figure 3:**
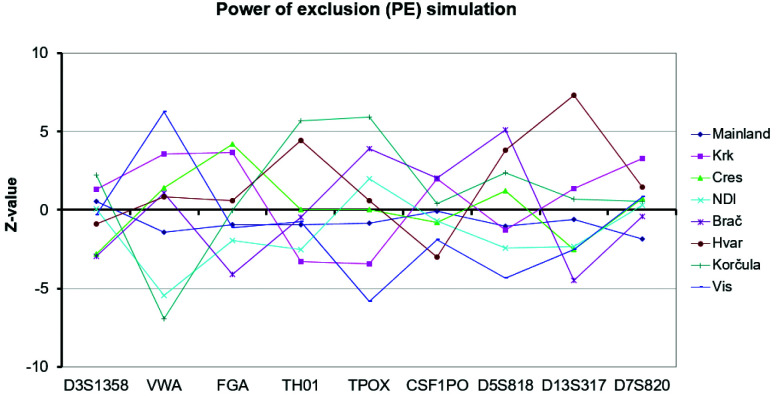
The power of exclusion (PE) Z-values in the subpopulations of Croatia in relation to the simulated population. NDI – North Dalmatian islands.

**Figure 4:**
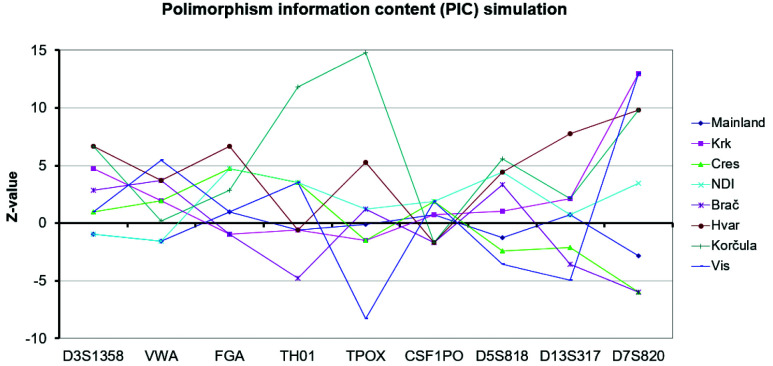
The polymorphic information content (PIC) Z-values in the subpopulations of Croatia in relation to the simulated population. NDI – North Dalmatian islands.

**Figure 5:**
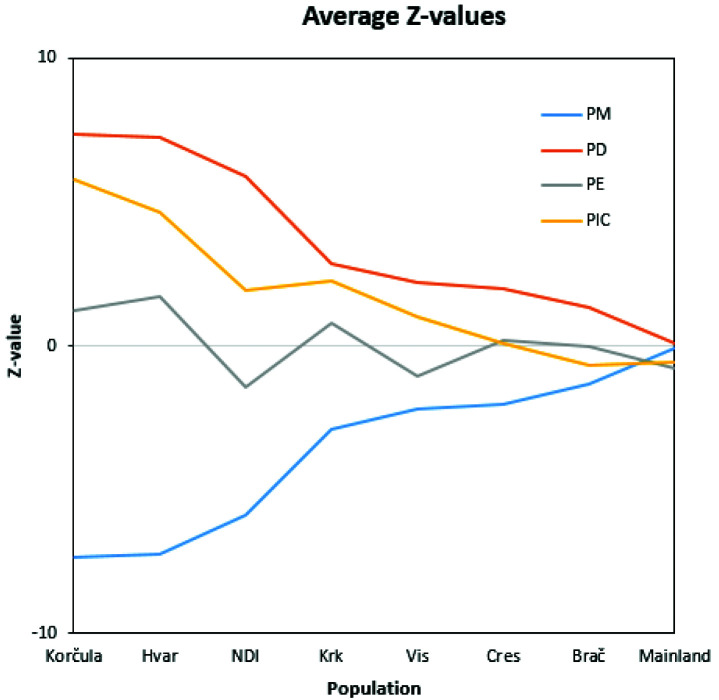
Average Z-values of individual forensic parameters (PM, PD, PE, PIC) for nine loci (D3S1358, vWA, FGA, TH01, TPOX, CSF1PO, D5S818, D13S317, D7S82) in the Croatian subpopulations.

The results of the forensic parameters analysis in eight different Croatian subpopulations are shown in Table 1 and Figures 1-5. The TPOX locus had the highest PM in all analyzed populations, based on the lowest level of heterozygosity, as well as the lowest PD and PE. On the other hand, the FGA locus was the most polymorphic locus in all Croatian populations, except on the island of Cres. It exhibited the highest H in most populations, except on Cres, Krk, Korčula, and NDI. It also showed the highest values of PD (with the exception of Cres) and PE (except on Krk, Korčula, and NDI).

**Table 1:** Forensic parameters of nine analyzed STR loci in populations of Croatia

Matching probability											
POPULATION	N	D3S1358	VWA	FGA	TH01	TPOX	CSF1PO	D5S818	D13S317	D7S820	REFERENCE
**continental Croatia**	149	0.074	0.063	0.038	0.09	0.207	0.138	0.117	0.081	0.068	Martinović Klarić et al. 2005 + current study (Baranja)
**Krk**	137	0.094	0.071	0.038	0.094	0.194	0.131	0.133	0.087	0.095	Martinović Klarić 2000
**Cres**	122	0.089	0.071	0.049	0.098	0.195	0.145	0.105	0.079	0.068	Novokmet et al. 2009
**North Dalmatian islands**	82	0.084	0.068	0.055	0.113	0.199	0.149	0.19	0.085	0.089	current study
**Brač**	96	0.095	0.077	0.042	0.09	0.22	0.117	0.139	0.065	0.061	Martinović Klarić 2000
**Hvar**	103	0.108	0.085	0.057	0.102	0.24	0.126	0.141	0.117	0.085	Martinović et al. 1999
**Korčula**	95	0.098	0.071	0.044	0.134	0.294	0.121	0.161	0.089	0.088	Martinović Klarić et al. 2001
**Vis**	98	0.085	0.075	0.043	0.099	0.16	0.153	0.117	0.065	0.093	Martinović Klarić et al. 2005

Power of discrimination											
POPULATION	N	D3S1358	VWA	FGA	TH01	TPOX	CSF1PO	D5S818	D13S317	D7S820	REFERENCE
**continental Croatia**	149	0.926	0.937	0.962	0.91	0.793	0.862	0.883	0.919	0.932	Martinović Klarić et al. 2005 + current study (Baranja)
**Krk**	137	0.906	0.929	0.962	0.906	0.806	0.869	0.867	0.913	0.905	Martinović Klarić 2000
**Cres**	122	0.911	0.929	0.951	0.902	0.805	0.855	0.895	0.921	0.932	Novokmet et al. 2009
**North Dalmatian islands**	82	0.916	0.932	0.945	0.887	0.801	0.851	0.81	0.915	0.911	current study
**Brač**	96	0.905	0.923	0.958	0.91	0.78	0.883	0.861	0.935	0.939	Martinović Klarić 2000
**Hvar**	103	0.892	0.915	0.943	0.898	0.76	0.874	0.859	0.883	0.915	Martinović et al. 1999
**Korčula**	95	0.902	0.929	0.956	0.866	0.706	0.879	0.839	0.911	0.912	Martinović Klarić et al. 2001
**Vis**	98	0.915	0.925	0.957	0.901	0.84	0.847	0.883	0.935	0.907	Martinović Klarić et al. 2005

Power of exclusion											
POPULATION	N	D3S1358	VWA	FGA	TH01	TPOX	CSF1PO	D5S818	D13S317	D7S820	REFERENCE
**continental Croatia**		0.525	0.693	0.725	0.56	0.293	0.438	0.507	0.539	0.638	Martinović Klarić et al. 2005 + current study (Baranja)
**Krk**	137	0.5	0.591	0.604	0.632	0.335	0.365	0.513	0.488	0.513	Martinović Klarić 2000
**Cres**	122	0.636	0.636	0.59	0.531	0.279	0.462	0.449	0.59	0.575	Novokmet et al. 2009
**North Dalmatian islands**	82	0.542	0.776	0.751	0.608	0.247	0.459	0.542	0.586	0.586	current study
**Brač**	96	0.642	0.642	0.808	0.546	0.216	0.364	0.35	0.642	0.603	Martinović Klarić 2000
**Hvar**	103	0.574	0.647	0.684	0.397	0.27	0.539	0.383	0.33	0.557	Martinović et al. 1999
**Korčula**	95	0.47	0.806	0.7	0.359	0.183	0.42	0.42	0.505	0.58	Martinović Klarić et al. 2001
**Vis**	98	0.554	0.536	0.729	0.554	0.374	0.501	0.591	0.591	0.573	Martinović Klarić et al. 2005

Polymorphism information content											
POPULATION	N	D3S1358	VWA	FGA	TH01	TPOX	CSF1PO	D5S818	D13S317	D7S820	REFERENCE
**continental Croatia**	149	0.76	0.8	0.84	0.74	0.56	0.66	0.69	0.75	0.78	Martinović Klarić et al. 2005 + current study (Baranja)
**Krk**	137	0.73	0.78	0.85	0.74	0.57	0.66	0.67	0.74	0.73	Martinović Klarić 2000
**Cres**	122	0.75	0.78	0.82	0.72	0.57	0.65	0.7	0.77	0.79	Novokmet et al. 2009
**North Dalmatian islands**	82	0.76	0.8	0.82	0.72	0.55	0.65	0.64	0.75	0.76	current study
**Brač**	96	0.74	0.77	0.85	0.76	0.55	0.68	0.65	0.78	0.79	Martinović Klarić 2000
**Hvar**	103	0.72	0.77	0.81	0.74	0.52	0.68	0.64	0.7	0.74	Martinović et al. 1999
**Korčula**	95	0.72	0.79	0.83	0.68	0.45	0.68	0.63	0.74	0.74	Martinović Klarić et al. 2001
**Vis**	98	0.75	0.76	0.84	0.72	0.62	0.65	0.71	0.79	0.73	Martinović Klarić et al. 2005

The number of observed alleles ranged from 5 at the loci D3S1358 (Krk and Korčula), TH01 (NDI), TPOX (Krk, Cres, Korčula, and NDI), and CSF (Vis) to 17 (FGA) in continental Croatia. The highest number of alleles was observed at FGA in all island populations (13 alleles for Hvar and Brač, 12 alleles for Krk, Cres, and Korčula, 11 alleles for NDI, and 10 alleles for Vis). Due to the largest number of alleles, the FGA locus had the highest H of all the analyzed loci in continental Croatia (0.865), on Brač (0.906), Hvar (0.845), and Vis (0.867). Although the largest number of alleles was observed in this same locus in other analyzed island populations, due to the presence of 4 (Krk, NDI, Korčula) to 5 (Cres) low-frequency alleles, this locus showed a lower H (0.803 for Krk, 0.795 for Cres, 0.878 for NDI, and 0.853 for Korčula), when compared with TH01 (0.818 for Krk), D3S1358 (0.820 for Cres), and VWA (0.890 for NDI, 0.905 for Korčula), for which a much smaller number of alleles was observed, but rarely with a frequency <2.5%.

If we consider PIC as the second measure of genetic marker informativeness (Table 1), the FGA locus was the most informative in the populations of continental Croatia, Brač, Hvar, and Vis, since it also showed the highest PIC – from 0.810 on Hvar to 0.850 on Brač. This locus also had the highest degree of polymorphism in the populations of Krk (0.850), Korčula (0.830), and NDI (0.820), but the highest H was not recorded. The population of Cres was the only population where the highest degree of the two analyzed measures was not observed at the FGA locus. On the other hand, the least informative genetic marker in all analyzed populations was the TPOX locus, as it showed the lowest H – from 0.495 (Korčula) to 0.663 (Vis), and the lowest PIC – from 0.450 (Korčula) to 0.620 (Vis).

### Forensic parameters in Southeast European populations

**Figure 6:**
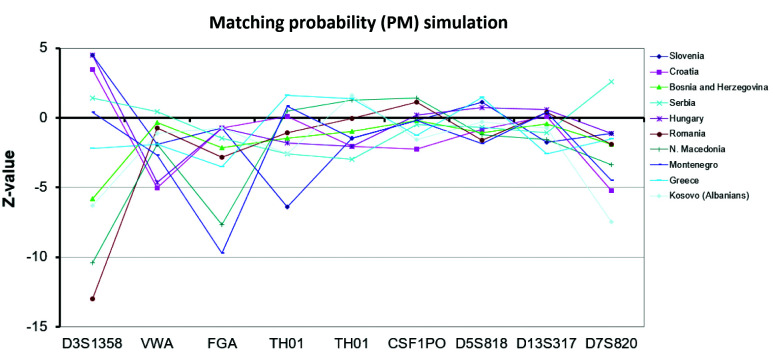
Matching probability (PM) Z-values in the populations of Southeastern Europe in relation to the simulated population. NDI – North Dalmatian islands.

**Figure 7:**
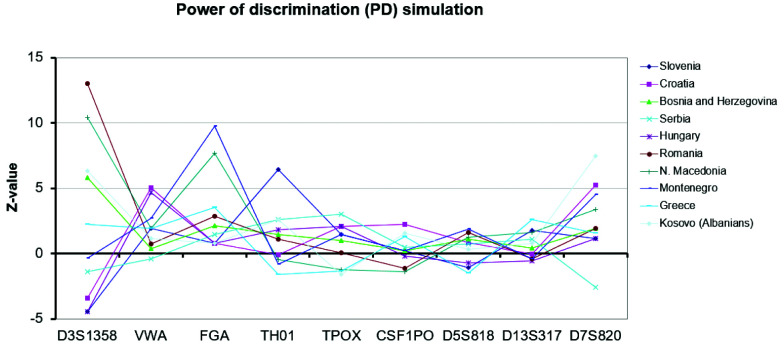
Power of discrimination (PD) Z-values in the studied populations of Southeastern Europe in relation to the simulated population.

**Figure 8:**
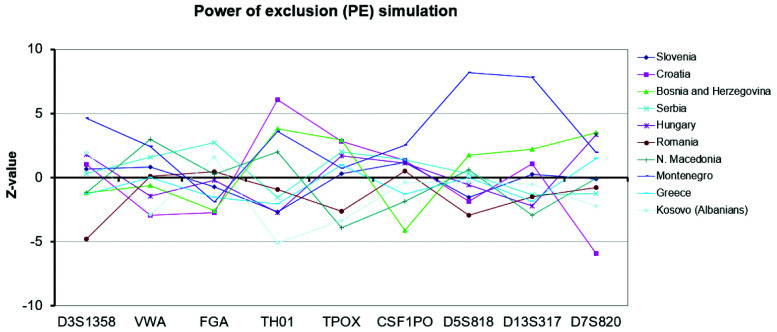
The power of exclusion (PE) Z-values in the studied populations of Southeastern Europe in relation to the simulated population.

**Figure 9:**
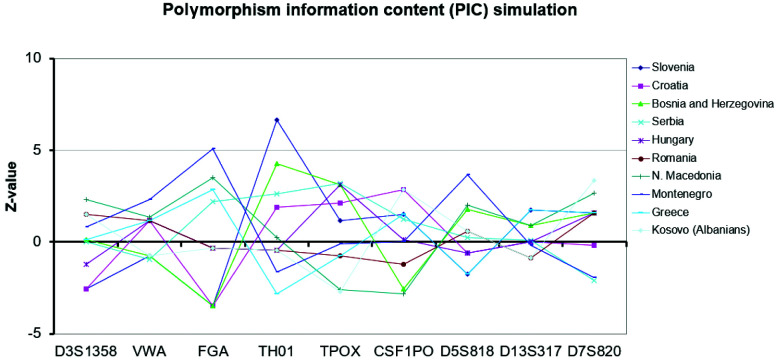
The polymorphic information content (PIC) Z-values in the studied populations of Southeastern Europe in relation to the simulated population.

**Figure 10:**
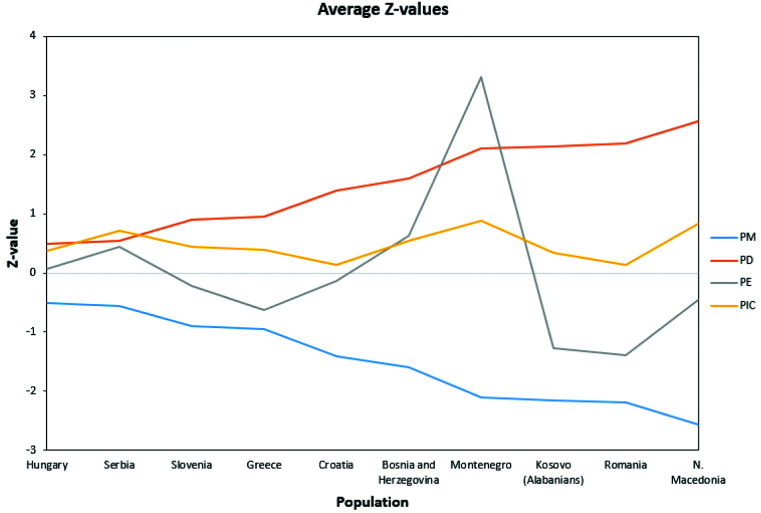
Average Z-values of individual forensic parameters (PM, PD, PE, PIC) for nine STR loci (D3S1358, vWA, FGA, TH01, TPOX, CSF1PO, D5S818, D13S317, D7S82) in the studied populations of Southeastern Europe.

The results of forensic parameters analysis in Southeast European populations are shown in Table 2 and Figures 6-10. The TPOX locus had the highest PM in all the analyzed populations (from 0.184 in Albanians from Kosovo to 0.223 in Serbia), based on the lowest H (with the exception of Montenegro) and PIC, and therefore also PD and PE. The only exception was the population of Montenegro, where this locus still retained the lowest PD, but not PE. Namely, the D5S818 locus had the lowest PE in this population, as well as the lowest H On the other hand, the FGA locus was the most polymorphic and heterogeneous locus in all populations. The only exceptions were the populations of Serbia, Romania, and Albanians from Kosovo. The highest H in the Serbian population was recorded at the D7S820 locus, in the Romanian population at D3S1358, and in the Kosovo Albanian population at VWA. These loci also had the highest degree of PE in these populations, unlike all other populations in which the highest PD and the PE were recorded at the FGA locus.

**Table 2:** Forensic parameters of nine analyzed STR loci in populations of Southeastern Europe

Matching probability											
Population	N	D3S1358	VWA	FGA	TH01	TPOX	CSF1PO	D5S818	D13S317	D7S820	REFERENCE
**Montenegro**	101	0.082	0.069	0.049	0.079	0.21	0.125	0.14	0.077	0.08	Jeran et al. 2007
**Serbia**	356	0.08	0.061	0.037	0.088	0.223	0.127	0.131	0.086	0.061	Novković et al. 2010
**North Macedonia**	100	0.103	0.067	0.046	0.08	0.187	0.113	0.135	0.089	0.077	Havaš et al. 2007
**Hungary**	223	0.074	0.074	0.036	0.086	0.215	0.122	0.12	0.076	0.071	Egyed et al. 2006
**Bosnia and Herzegovina**	100	0.094	0.063	0.038	0.085	0.206	0.125	0.134	0.082	0.073	Marjanović et al. 2006
**Romania**	222	0.108	0.064	0.039	0.084	0.198	0.115	0.138	0.077	0.073	Marian et al. 2006
**Albanians from Kosovo**	137	0.095	0.065	0.036	0.088	0.184	0.135	0.128	0.084	0.088	Kubat et al. 2004
**Greece**	297	0.087	0.067	0.04	0.077	0.186	0.133	0.114	0.095	0.072	Sánchez-Diz et al. 2008
**Slovenia**	111	0.074	0.067	0.036	0.098	0.21	0.125	0.117	0.09	0.071	current study

Power of discrimination											
Population	N	D3S1358	VWA	FGA	TH01	TPOX	CSF1PO	D5S818	D13S317	D7S820	REFERENCE
**Montenegro**	101	0.918	0.931	0.951	0.921	0.79	0.875	0.86	0.923	0.92	Jeran et al. 2007
**Serbia**	356	0.92	0.939	0.963	0.912	0.777	0.873	0.869	0.914	0.939	Novković et al. 2010
**North Macedonia**	100	0.897	0.933	0.954	0.92	0.813	0.887	0.865	0.911	0.923	Havaš et al. 2007
**Hungary**	223	0.926	0.926	0.964	0.914	0.785	0.878	0.88	0.924	0.929	Egyed et al. 2006
**Bosnia and Herzegovina**	100	0.906	0.937	0.962	0.915	0.794	0.875	0.866	0.918	0.927	Marjanović et al. 2006
**Romania**	222	0.892	0.936	0.961	0.916	0.802	0.885	0.862	0.923	0.927	Marian et al. 2006
**Albanians from Kosovo**	137	0.905	0.935	0.964	0.912	0.816	0.865	0.872	0.916	0.912	Kubat et al. 2004
**Greece**	297	0.913	0.933	0.96	0.923	0.814	0.867	0.886	0.905	0.928	Sánchez-Diz et al. 2008
**Slovenia**	111	0.926	0.933	0.964	0.902	0.79	0.875	0.883	0.91	0.929	current study

Power of exclusion											
**Population**	**N**	**D3S1358**	**VWA**	**FGA**	**TH01**	**TPOX**	**CSF1PO**	**D5S818**	**D13S317**	**D7S820**	**REFERENCE**
**Montenegro**	101	0.464	0.531	0.757	0.531	0.32	0.403	0.23	0.433	0.566	Jeran et al. 2007
**Serbia**	356	0.579	0.559	0.626	0.632	0.289	0.441	0.454	0.559	0.648	Novković et al. 2010
**North Macedonia**	100	0.618	0.51	0.695	0.562	0.428	0.545	0.444	0.581	0.618	Havaš et al. 2007
**Hungary**	223	0.539	0.664	0.708	0.655	0.297	0.449	0.478	0.571	0.531	Egyed et al. 2006
**Bosnia and Herzegovina**	100	0.618	0.637	0.775	0.527	0.268	0.618	0.413	0.51	0.527	Marjanović et al. 2006
**Romania**	222	0.715	0.611	0.689	0.619	0.398	0.468	0.545	0.561	0.636	Marian et al. 2006
**Albanians from Kosovo**	137	0.535	0.715	0.658	0.701	0.415	0.497	0.473	0.548	0.672	Kubat et al. 2004
**Greece**	297	0.622	0.615	0.746	0.642	0.314	0.526	0.462	0.566	0.578	Sánchez-Diz et al. 2008
**Slovenia**	111	0.569	0.586	0.724	0.654	0.329	0.447	0.506	0.537	0.619	current study

Polymorphism information content											
**Location**	**N**	**D3S1358**	**VWA**	**FGA**	**TH01**	**TPOX**	**CSF1PO**	**D5S818**	**D13S317**	**D7S820**	**REFERENCE**
**Montenegro**	101	0.745	0.774	0.833	0.765	0.573	0.681	0.644	0.752	0.79	Jeran et al. 2007
**Serbia**	356	0.751	0.791	0.842	0.747	0.539	0.672	0.673	0.749	0.791	Novković et al. 2010
**North Macedonia**	100	0.734	0.779	0.838	0.757	0.599	0.702	0.658	0.74	0.764	Havaš et al. 2007
**Hungary**	223	0.76	0.78	0.85	0.76	0.54	0.68	0.68	0.75	0.77	Egyed et al. 2006
**Bosnia and Herzegovina**	100	0.75	0.79	0.86	0.74	0.54	0.7	0.66	0.74	0.77	Marjanović et al. 2006
**Romania**	222	0.74	0.78	0.85	0.76	0.58	0.69	0.67	0.76	0.77	Marian et al. 2006
**Albanians from Kosovo**	137	0.74	0.79	0.85	0.76	0.6	0.66	0.67	0.76	0.76	Kubat et al. 2004
**Greece**	297	0.75	0.78	0.84	0.77	0.58	0.67	0.69	0.73	0.77	Sánchez-Diz et al. 2008
**Slovenia**	111	0.77	0.79	0.86	0.73	0.56	0.67	0.69	0.73	0.77	current study

The informativeness of the nine analyzed STR loci was assessed by using the same two measures as for the Croatian subpopulations, H and PIC. The FGA locus had the highest H in most of the analyzed populations. Although the highest number of alleles was observed in this same locus in all analyzed populations, due to the presence of 2 (population of Romania) to 7 (populations of Serbia and Albanians from Kosovo) low-frequency alleles, this locus in some populations showed a lower H. If we take into account H and PIC (Table 2), the FGA locus was the most informative in Montenegro, North Macedonia, Hungary, Bosnia and Herzegovina, Greece, and Slovenia, exhibiting the highest H and PIC (from 0.860 in Bosnia and Herzegovina and Slovenia to 0.833 in Montenegro). The highest PIC was also observed at this same locus in all other analyzed populations (populations of Serbia, Romania, and Albanians from Kosovo), but without the highest degree of heterozygosity. On the other hand, the TPOX locus was the least informative as it showed the lowest degree of polymorphism in all analyzed populations (from 0.539 in Serbia to 0.600 in Albanians from Kosovo) and the lowest H (from 0.580 in Bosnia and Herzegovina to 0.700 North Macedonia) in all populations, except in the population of Montenegro, where the lowest value of H was measured at the D5S818 locus.

### Z-values of the analyzed forensic parameters in Croatian subpopulations

The largest deviations of PM were observed at the loci TH01 and TPOX among participants from Korčula, and at the locus TPOX in the population of Vis (Figure 1). The largest deviations of PD were observed at the TH01 and TPOX loci among participants from Korčula, followed by the population of Hvar, where deviations were observed at the D3S1358 and FGA loci. Deviations at the TPOX and D7S820 loci were observed in the Vis population, while deviations at the FGA, TH01, and D5S818 loci were observed in the NDI population (Figure 2). The smallest and most uniform deviations were determined for PE (Figure 3). However, when inspecting the observed deviations in detail, we observed that they were again largest in the populations of Korčula, Hvar, and Vis. The largest deviations of the PIC were again shown at the TPOX and TH01 loci in the population of Korčula, at the TPOX locus in the population of Vis, and at the D7S820 locus in the population of Krk and Vis (Figure 4).

The largest average values of the four analyzed forensic parameters in the studied Croatian subpopulations were found in the population of Korčula, followed by the population of Hvar. The smallest deviations were found in the mainland population (Figure 5).

### Z-values of analyzed forensic parameters in Southeast European populations

The largest deviations of PM were observed at the D3S1358 locus in the populations of Romania and North Macedonia, and at the FGA locus in the population of Montenegro (Figure 6). The largest deviations of PD were observed at the D3S1358 locus in the populations of Romania and North Macedonia and at the FGA locus in the population of Montenegro (Figure 7). The largest deviations of PE were found at the loci D5S818 and D13S317 in the population of Montenegro, and the loci TH01 and D7S820 in the population of Croatia (Figure 8). The largest deviations of the PIC degree of polymorphism were found at the TH01 locus in the population of Slovenia, followed by the FGA locus in the population of Montenegro, Slovenia, Bosnia and Herzegovina, and Croatia (Figure 9).

The average values of the four analyzed forensic parameters for all nine loci in the studied Southeast European populations are shown in Figure 10. The largest deviations were found in the population of North Macedonia, followed by Romania, Albanians from Kosovo, and Montenegro.

## Discussion

In our study, based on the conducted informativeness analyses of STR loci, the FGA locus was the most informative, while the TPOX locus was the least informative locus in most analyzed Croatian subpopulations. This finding confirms the results of previous research (9), which determined the highest genetic diversity (heterozygosity) for the FGA locus (83.9%-86.9%), and the lowest for the TPOX locus (50.9%-67.6%). Similar findings for these two loci have been confirmed in Southeast European populations and other similar studies (8,27).

### Influence of interpopulation relations on forensic parameters of STR markers

Although the degree of total genetic differentiation (FST) for the studied Croatian subpopulations was low, which indicates low substructuring, the difference between individual population groups cannot be ignored and should be taken into account when creating an appropriate population DNA database. Bearing in mind the structural complexity of Croatia, whose subpopulations still retain their distinctive features shaped by different ethno-historical migratory processes and lifestyles, it is necessary to determine the impact of small but significant substructuring levels in particular populations (28).

Croatia has numerous island populations with pronounced endogamy practices, which in small communities can lead to consanguinity (29). Diversity caused by substructuring due to isolation and blood relationship may be emphasized in specific populations, and may be important for forensic evaluation of DNA profiles (30,31). If such relationships exist within the population, it is necessary to develop more precise population DNA databases. In order to help create a DNA database that would best represent the substructuring of the Croatian population, we compared the forensic parameters of genetic STR loci calculated for each population group with a potential reference base obtained by random selection from the analyzed data. The largest deviations were observed in the population of the island of Korčula, followed by the island of Hvar. Since the previously described methods suggested that the population of Korčula was the most closed and isolated, the deviation of this population indicates that substructuring influenced the forensic parameters of the studied STR markers. On the other hand, the smallest deviations from the “random sample” were observed in the mainland population, as expected. The obtained results indicate the specificity of individual populations that should be taken into account when creating a reference DNA database of all subpopulations in Croatia. Such a database would be used to determine the frequency of alleles of different loci and to statistically calculate the “rarity” of a particular DNA profile within the general population (32,33).

The results of the forensic parameters analysis in all studied Croatian and Southeast European populations suggest the lowest informativeness of the TPOX locus, which had the highest PM in all analyzed populations, as well as the lowest H and PIC. On the other hand, the most informative locus was the FGA. The FGA was the most polymorphic locus with the highest degree of heterozygosity, as well as discriminant power and PE, and the lowest values of PM in most populations. The standard index of genetic diversity, as well as genetic diversity at the molecular level, also determined the lowest values at the TPOX and the highest at the FGA locus.

The average values of basic forensic parameters for all nine loci showed greater deviations at the level of Croatian subpopulations than at the level of Southeast European populations. In Croatia, the largest deviations were found in the population of the Korčula, followed by the population of Hvar, while the smallest deviations were found in the mainland population. Among Southeast European populations, the largest deviations were observed in the population of North Macedonia, followed by the populations of Romania, Albanians from Kosovo, Montenegro, Bosnia and Herzegovina, and Croatia.

The comparison of forensic parameters between different subpopulations of Croatia and Southeast Europe indicates that the isolation of individual Croatian subpopulations and certain rare alleles in their gene pool affect the values of forensic parameters. This should be taken into account in the sampling of the total population when creating a DNA database of STR markers. Individuals from such isolates must be represented in sufficient numbers to comply with the above rule, and rare alleles characteristic of Croatian subpopulations were included in the representative base used in this article. The main limitation of our study was using only 9 STR loci, because it was the usual available raw data. Furthermore, we were limited to the selected population data from the Southeastern European region that were published at the time and could not use all the population data. However, our results confirm that the influence of structuring of the two hierarchal levels assessed in our research could be determined with this number of loci. Additional research should further explain the influence of more STR of loci on substructuring, as well as perform the comparison with larger population data sets.
